# Supplementary material for: Comprehensive Annotation and Functional Exploration of MicroRNAs in Lettuce
Source: Front Plant Sci. 2021 Dec 24;12:781836. doi: 10.3389/fpls.2021.781836 (PMC8739914; doi:10.3389/fpls.2021.781836)
Supplement: Supplementary file 2 [file Data_Sheet_8.ZIP › GSM2230748.plot/Lsa-miRN533_Lsat_1_v5_gn_5_38921.1_537_TPlot.pdf]

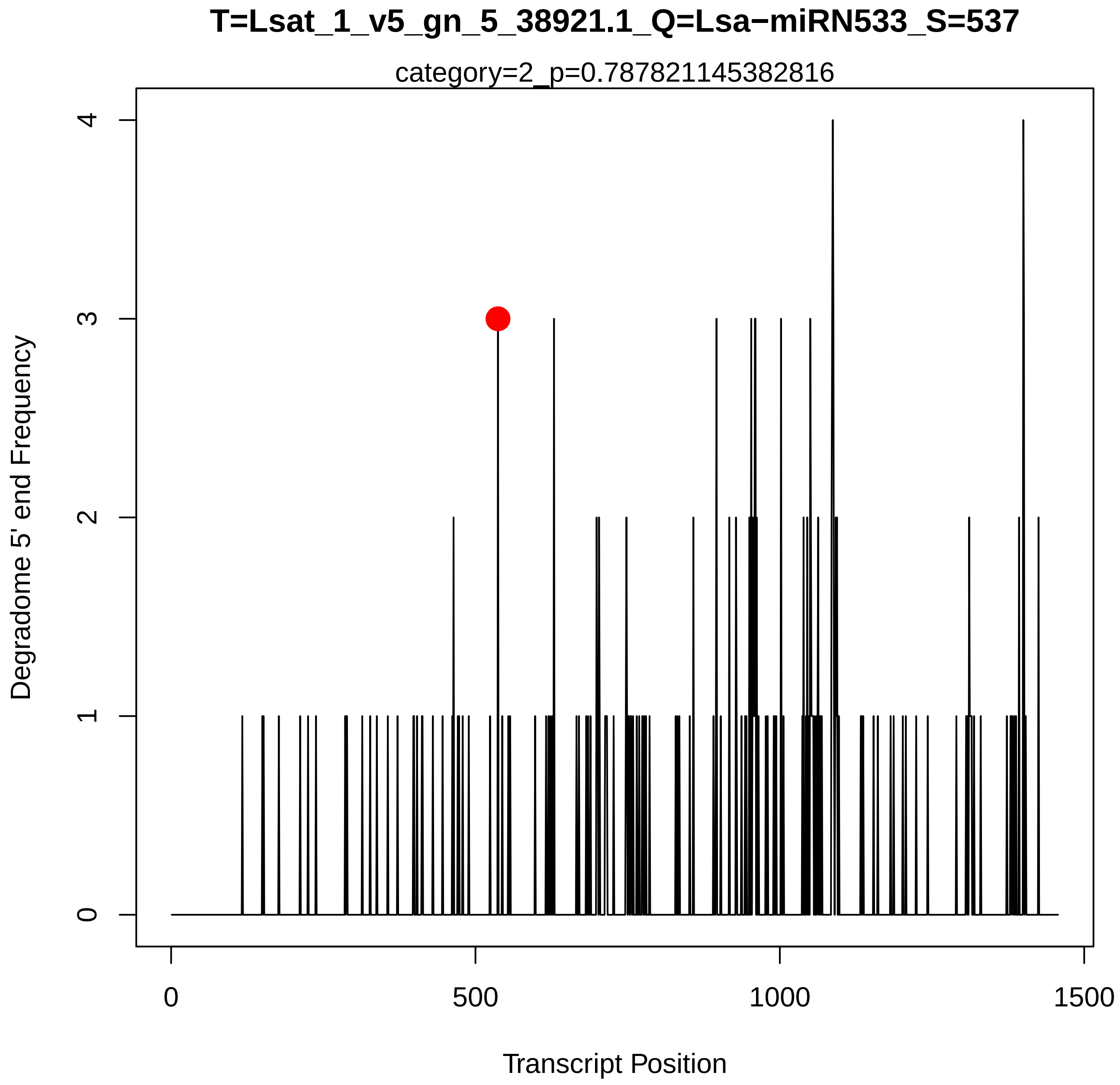

T=Lsat_1_v5_gn_5_38921.1_Q=Lsa−miRN533_S=537
category=2_p=0.787821145382816
Degradome 5' end Frequency
Transcript Position
0
1
2
3
4
0
500
1000
1500
